# Effects of SF_6_ decomposition components and concentrations on the discharge faults and insulation defects in GIS equipment

**DOI:** 10.1038/s41598-020-72187-0

**Published:** 2020-09-14

**Authors:** Yuan Zhuang, Xiaotong Hu, Bin Tang, Siwei Wang, Anyang Cui, Keyong Hou, Yunhua He, Liangqing Zhu, Wenwu Li, Junhao Chu

**Affiliations:** 1 Technical Center for Multifunctional Magneto-Optical Spectroscopy (Shanghai), School of Communication and Electronic Engineering, East China Normal University, Shanghai, 200241 China; 2 Electric Power Research Institute, Guangxi Power Grid Co. Ltd., Nanning, 530023 China; 3 Key Laboratory of Separation Science for Analytical Chemistry, Dalian Institute of Chemical Physics, Chinese Academy of Sciences, Dalian, 116023 China; 4 Electric Power Research Institute, Yunnan Power Grid Co. Ltd., Kunming, 650217 China

**Keywords:** Electrical and electronic engineering, Atomic and molecular physics, Materials chemistry, Theoretical chemistry

## Abstract

Gas-insulated switchgear (GIS) is widely used across multiple electric stages and different power grid levels. However, the threat from several inevitable faults in the GIS system surrounds us for the safety of electricity use. In order to improve the evaluation ability of GIS system safety, we propose an efficient strategy by using machine learning to conduct SF_6_ decomposed components analysis (DCA) for further diagnosing discharge fault types in GIS. Note that the empirical probability function of different faults fitted by the Arrhenius chemical reaction model has been investigated into the robust feature engineering for machine learning based GIS diagnosing model. Six machine learning algorithms were used to establish models for the severity of discharge fault and main insulation defects, where identification algorithms were trained by learning the collection dataset composing the concentration of the different gas types (SO_2_, SOF_2_, SO_2_F_2_, CF_4_, and CO_2_, etc.) in the system and their ratios. Notably, multiple discharge fault types coexisting in GIS can be effectively identified based on a probability model. This work would provide a great insight into the development of evaluation and optimization on solving discharge fault in GIS.

## Introduction

Sulfur hexafluoride (SF_6_) is widely used in Gas-Insulated Switchgear (GIS) because of its excellent insulation property, heat dissipation, and arc extinguishing performance. On the other hand, owing to the subtle insulation defects in the manufacturing process, discharge faults will be caused during the operation of the GIS equipment. As a result, SF_6_ will be decomposed into lower sulfur fluorides under discharge induced by insulation defects^1,2^. These products will threaten the safety of the entire power grid system. When the SF_6_ decomposes into other products under discharge, the insulating performance of GIS will be reduced. It will further threaten the safety of GIS equipment. Although SF_6_ is non-toxic, some SF_6_ decomposition products are toxic such as S_2_F_10_, SF_4_, SOF_2_, SO_2_F_2_, SOF_4_, and HF, which will threaten the GIS equipment and environmental protection^3,4^. Therefore, an effective pre-diagnosis method for discharge faults is necessary for the current industrial GIS system to recognize and maintain insulation defects. However, owing to the complexity of the equipment and the numerous factors of discharge fault, these internal defects are generally difficult to be recognized and further maintained.

One of the key parameters for effectively evaluating the discharge fault is the SF_6_ decomposed components. Many detection techniques have been applied to the analysis of SF_6_ decomposition products, such as gas chromatography, gas detection tubes, electrochemical methods, and spectral methods^5^. Moreover, various decomposition components, such as CF_4_, CO_2_, SOF_2_, and SO_2_F_2_, can effectively reflect the severity of the discharge fault and the type of insulation defects^6,7^. Since the relationship between SF_6_ decomposition products and electrical faults is quite complicated, the discharge fault classification obtained by traditional component analysis is not reliable enough. While the use of machine learning can clarify the numerical boundaries of different fault types in the data field, which will facilitate the application in automation. Therefore, it is significant to systematically study the data of decomposition products by machine learning training based on the Decomposed Components Analysis (DCA) method, and further obtain an identification model for discharge fault. Wang et al*.* used an Adaptive Fuzzy Neural Inference System (AFNIS) to identify four Partial Discharge (PD) faults^8^. Tang et al*.* summarized the physical meanings of three characteristic parameters CF_4_/CO_2_, SOF_2_/SO_2_F_2,_ and (SOF_2_ + SO_2_F_2_)/(CF_4_ + CO_2_). These characteristic parameters are suitable for Support Vector Machines (SVM) to detect the type of insulation fault under PD^9^. Ding et al*.* found that corona discharge and spark discharge can be distinguished by testing the concentration ratio of (SOF_2_ + SO_2_)/(SO_2_F_2_)^10^. Their experiment indicated that the (SOF_2_ + SO_2_)/(SO_2_F_2_) concentration ratio of corona discharge generally ranges from 0 to 1, while that of spark discharge ranges from 1 to 5. Although many effective methods and parameters have been applied to identify the discharge faults in GIS equipment, the machine learning dataset in most of the researches is limited to one series of experiments. Such a specific environment will make it difficult to reach a general conclusion. In addition, an identification model based on various environmental data and an optimal algorithm is lacking. More importantly, the coexistence state of multiple insulation defects in the discharge fault is currently short of the identification model^11,12^.

In this paper, four main insulation defect types (particle, pollution, gap, protrusion) were taken into consideration. These insulation defects will gradually lead to three severity types of the fault discharge (corona, spark, arc). These two types of identification based on SF_6_ decomposition component will be helpful for rapid diagnosis of the cause and condition of fault discharge. First, a large amount of discharge fault data from different experiments was analyzed. Then, two types of functions were used to preprocess the data. The empirical probability functions were fitted according to the Arrhenius model of a chemical reaction and the characteristic of the data distribution, while the tensile functions derived from experience for stretching data. After data preprocessing with these functions, various machine learning algorithms were used and compared to obtain a robust and reliable model to describe the relationship between the feature components of SF_6_ decomposition and discharge fault. As a result, the severity of the discharge fault can be determined by SO_2_F_2_/SO_2_ in the K-Nearest Neighbor (KNN) model. The insulation defects under PD can be described by the parameters (SOF_2_ + SO_2_F_2_)/(CF_4_ + CO_2_) and CF_4_/CO_2_ in the Gaussian Distribution model. Based on the probability functions used in both models, two coexistence states of multiple insulation defects, biased corona discharge state, and surface pollution defects can also be recognized. These findings would provide new insight into handling the actual problems in the discharge fault and further promoting the development of the relevant GIS system.

## Methods

**Figure 1:**
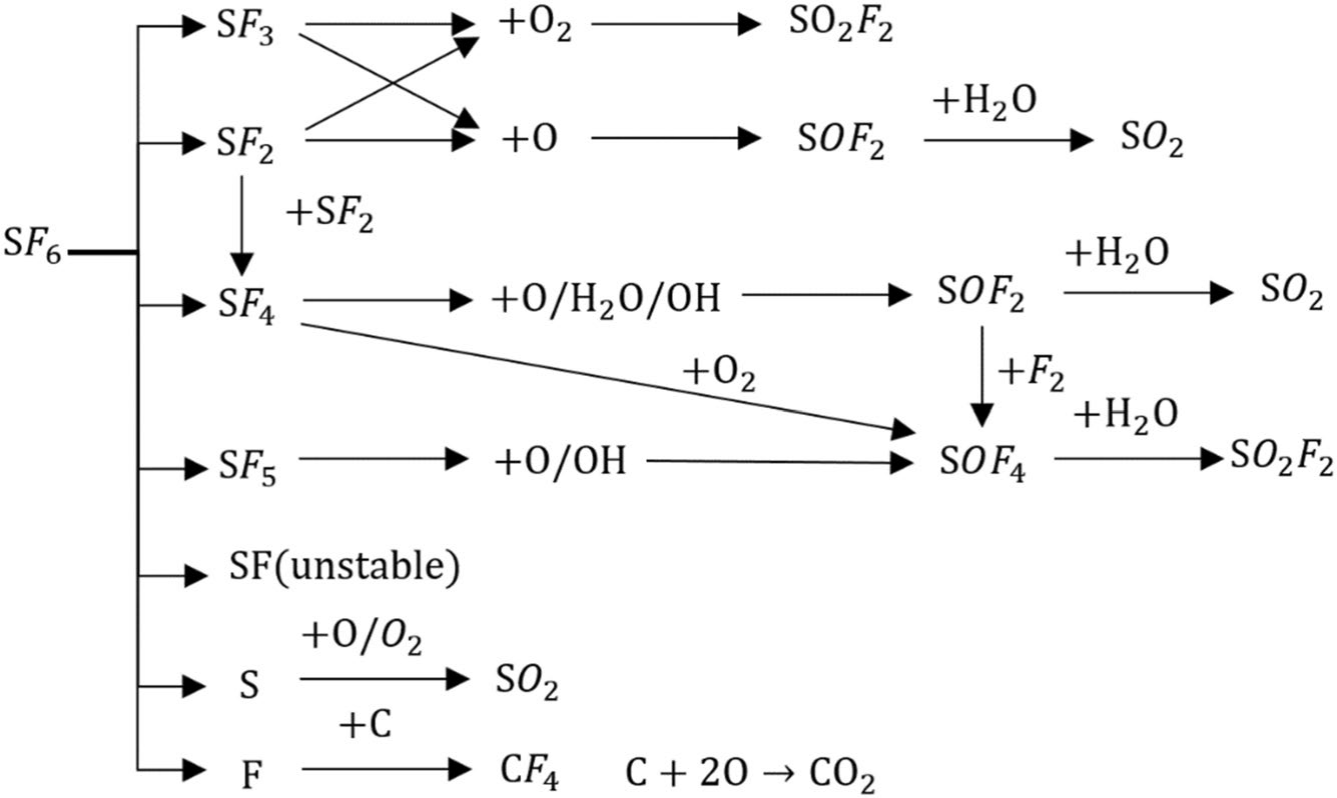
The main chemical reaction process for the decomposition products of SF_6_.

The decomposition of SF_6_ courses with the breaking of S-F chemical bonds and the fractured bond number is proportional to the discharge energy. Although more energy is required for breaking S-F bonds, SF_4_ and SF_2_ are the main decomposition products of SF_6_, because they are more stable than SF_5_ and SF_3_ with their symmetrical structure. The SF_5_, SF_3_, SF products are easy to combine with the free F atoms, so they are not stable. Furthermore, the secondary ionization rate of SF_x_ under the reaction of partial discharge(PD) is very small. This means that the decomposition products of SF_6_ will mainly be obtained by the first ionization^13–15^. Owing to the existence of H_2_O and O_2_ in GIS, the SF_6_ decomposition products further react with H_2_O and O_2_, thus making the decomposition more complex, such as SOF_2_, SO_2_F_2_, and SO_2_^7,16^. In addition, carbon atoms presenting in the gas chamber will react with the element F and O to form CF_4_ and CO_2_. These main possible split process in GIS can be represented by the formula (1) and Fig. 1.

1 $${e}^{-}+{SF}_{6}\to {SF}_{x}+(6-X)F+{e}^{-}, X=1, 2, 3, 4, 5$$

Compared with the simple reaction of SF_2_ and SF_3_, the process of SF_4_ and SF_5_ reacting to generate characteristic gases SOF_2_, SO_2_F_2,_ and SO_2_ is complicated with more intermediates referred. This further indicates that it is hard to identify the decomposition process of SF_6_ from the decomposition mechanism, because of the various source of the characteristic gas, together with the variable complexity of different ways. Considering the complex decomposition process of SF_6_, DCA, a method for analyzing the discharge faults according to the SF_6_ decomposition, cannot identify the discharge fault type efficiently. Instead, machine learning is a wise strategy to train the identification model. In this model, severity and insulation defects of discharge fault need to be chosen as the targets, which are important for GIS system to maintain the insulation defects timely.

### Data collection and usage instructions

The discharge faults originate from the various insulation defects in GIS devices. These defects mainly include four types: protrusion, particle, pollution, and gap. The manifestations of these discharge faults will also show different stages, which can be roughly divided into three categories: corona discharge (PD), spark discharge, and arc discharge. These two types of identification based on SF_6_ decomposition component will be helpful for rapid diagnosis of the cause and condition of fault discharge. In this work, the SF_6_ decomposition data from real GIS system was gathered by infrared absorption spectrometry and gas chromatography^17,18^. Then, the data recorded per 12 h from 24 to 72 h after the fault occurs were selected into the sample set. As a result, a total of 222 samples were used for training, and 24 samples were used for testing. Most of them were collected from laboratory simulations and field cases of the published articles, while the others were provided by China Southern Power Grid from the field measurements of GIS faults. Also, all machine learning algorithms share this data set. The data collection and processing are summarized in Table 1. Furthermore, in order to clarify the composition of the data, Table 2 has listed the types of failures involved in our research and the corresponding amounts of training data and test data.

**Table 1:** The selected analytical methods, decomposition products, and time delay for different discharge fault analysis

Fault types	Analytical methods	Data	Time delay
Severity types (arc, spark, corona)	Six machine learning methods (Neural Network, SVM, Linear Regression, K-Nearest Neighbor, Random Forest, and Gaussian Distribution)	SO_2_F_2_, SO_2_	Per 12 h (from 24 to 72 h)
Insulation defect types (particle, pollution, gap, protrusion)		SOF_2_, SO_2_F_2_, CF4, CO_2_

**Table 2:** The relationship among the fault types, training data, and testing data

	Fault type	Number of training data	Number of test data
Severity type	Arc discharge	12	0
	Spark discharge	28	4
	Corona discharge	21	3
Insulation defect type	Protrusion defect	48	7
	Particle defect	18	2
	Pollution defect	47	4
	Gap defect	48	4

### Identification model for severity of discharge fault

The SF_6_ decomposition component data for training are from the faulted GIS equipment (Guangxi Power Grid Co. Ltd.) and the relative references^11,19–23^. Some data from other references were also collected to construct a testing dataset^11,23,24^. The type of severity of the discharge fault in GIS equipment can be expressed according to the energy level of internal discharge faults, which are divided into corona discharge (low), spark discharge (middle), and arc discharge (high). According to the level of energy, the arc discharge was labeled as a single scalar of 3, the spark discharge was labeled as 2, and the corona discharge was labeled as 1. Based on the traditional DCA method, the ratio of the concentrations of SO_2_F_2_ and SO_2_ was regarded as the effective features to represent the severity of the discharge fault^12,25,26^. In order to make sure and further quantify the relationship between the severity of discharge fault and concentration ratio of SO_2_F_2_/SO_2_, six different algorithms (Neural Network, SVM, Linear Regression, K-Nearest Neighbor, Random Forest, and Gaussian Distribution) were employed to learn the data for effectively recognizing the discharge fault type. However, without considering the coexistence of multiple discharge faults, the original model fails to provide a good identification model. In this model, a clear boundary will always exist between the two discharge fault states. Such a steep boundary is easy to cause misjudgment.

To improve the prediction ability of models, the empirical probability functions (2)–(4) listed below are required to assign different weights to the data for machine learning. These empirical probability functions were obtained based on statistic point analyzation and the Arrhenius model of a chemical reaction. Details on how to induce these functions will be discussed in the results. In these functions, the variable $x$ represents SO_2_F_2_/SO_2_, while the dependent variable $f(x)$ means different weights for different values of SO_2_F_2_/SO_2_.

2 $${f}_{1}\left(x\right)=-4{x}^{2}+1(0\le x\le 0.5)$$

3 $${f}_{2}\left(x\right)=\frac{0.6}{1+{e}^{4\times (3.7-x)}}+\frac{0.4}{1+{e}^{0.5\times \left(8-x\right)}}\left(x\ge 0.5\right)$$

4 $${f}_{3}\left(x\right)=1-{f}_{1}-{f}_{2}$$

**Figure 2:**
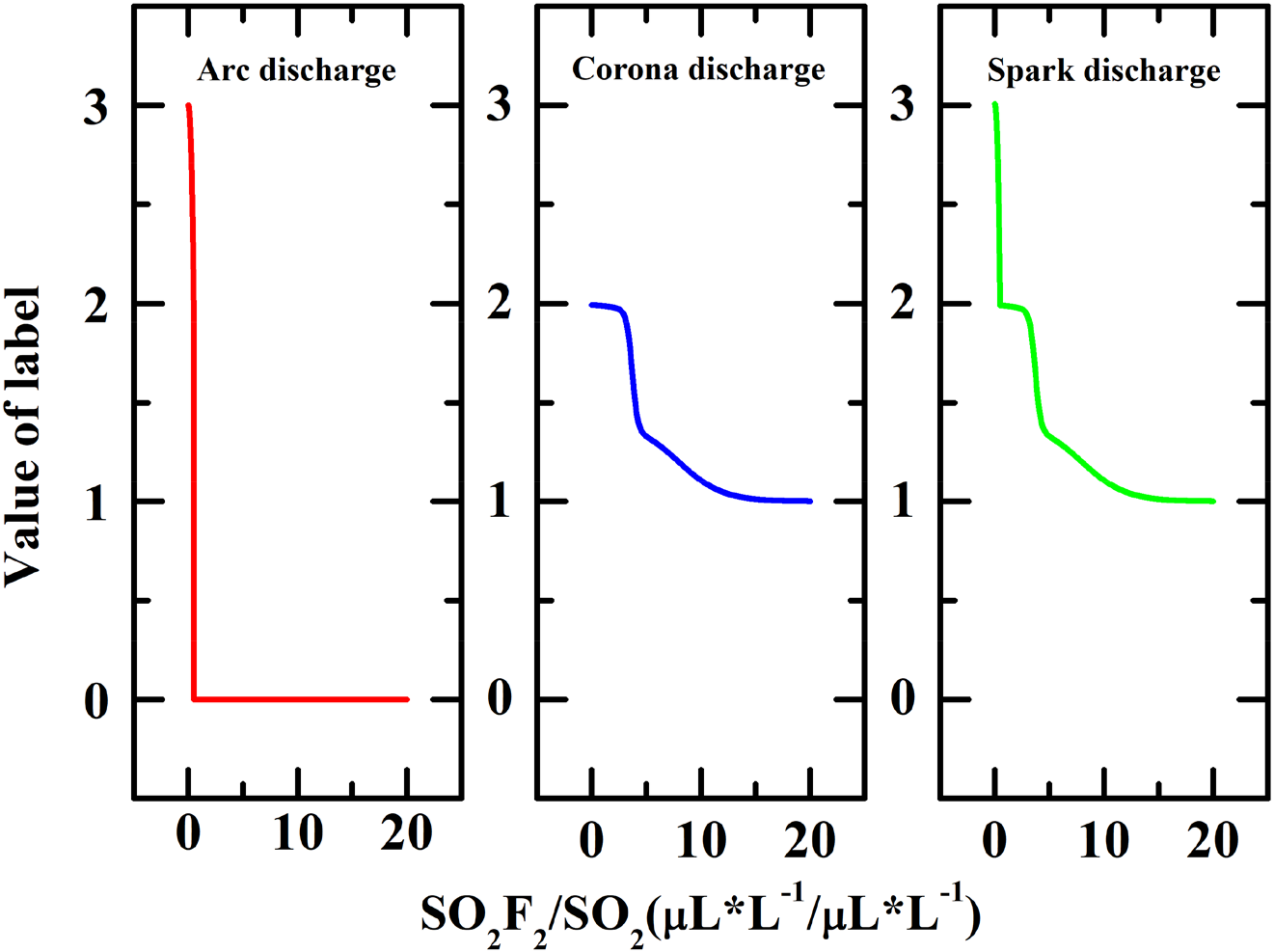
The empirical probability functions used to adjust the original labels 3, 2, 1 of the three discharge types.

Then the empirical probability functions were used to adjust the original label 3, 2, 1 of the three discharges data: arc discharge data corresponds to the value of $2+{f}_{1}(x)$, while spark discharge data corresponds to the value of $2-{f}_{2}(x)$. The corona discharge data corresponds to the value of $1+{f}_{3}(x)(0.5\le x)$*,*$3-{f}_{3}(x)(x<0.5)$*.* These corresponding relations between the discharge type and label value are shown in Fig. 2.

After pretreating the feature of the sample data, models with better recognition ability could be obtained by machine learning. Finally, KNN was chosen to establish the discharge fault severity model and tested by the testing dataset prepared before.

### Identification model for insulation defects of discharge fault

With a similar process, SF_6_ decomposition component data from another group of references were collected to create the identification model for insulation defects of discharge fault^8,12,27–31^. Some of them were isolated to form test datasets^12,31^. The types of insulation defects of the discharge fault can be divided into four main categories including particle defect (labeled as 1), pollution defect (labeled as 2), gap defect (labeled as 3), and protrusion defect (labeled as 4). Three characteristic parameters, CF_4_/CO_2_, SOF_2_/SO_2_F_2,_ and (SOF_2_ + SO_2_F_2_)/(CF_4_ + CO_2_), can be considered as the typical features in order to better distinguish these four insulation defects^6^. However, the parameter SOF_2_/SO_2_F_2_ should not be used as a characteristic ratio^32–35^. Because the GIS internal adsorbent has changeable absorption rates for different SF_6_ decomposed products, thus making the parameter SOF_2_/SO_2_F_2_ more dispersive. In addition, the difference in parameter SOF_2_/SO_2_F_2_ between the overheat faults and discharge faults is not obvious. This makes the use of this parameter is not conducive for overheating fault to be distinguished from the discharge fault in further applications.

In the case of selecting CF_4_/CO_2_ and (SOF_2_ + SO_2_F_2_)/(CF_4_ + CO_2_) as the features, the data points were distributed on a two-dimensional plane. The x coordination is represented by function (5), while the y coordination is represented by function (6). Considering the data points in a two-dimensional distribution, it’s hard to fit empirical probability functions. Instead, tensile functions (5)–(6) were used to extend the steep boundary between data points of different insulation defects. This adjustment allows the data of insulation defect to have comparable value on the coordinate axes of the characteristic parameters (SOF_2_ + SO_2_F_2_)/(CF_4_ + CO_2_) and CF_4_/CO_2_, which is of great significance for machine learning training.

Regarding the characteristic parameters (SOF_2_ + SO_2_F_2_)/(CF_4_ + CO_2_), plenty of studies have shown that when the process of discharge involves overheating faults and solid insulation materials, the production of CF_4_ and CO_2_ will be less than various sulfur containing characteristic products. Therefore, it can be found that the characteristic parameters (SOF_2_ + SO_2_F_2_)/(CF_4_ + CO_2_) of the partial discharge samples are very large. In general, the characteristic parameter lg(SOF_2_ + SO_2_F_2_)/(CF_4_ + CO_2_) would be applied instead. However, it is worth noting that if the organic insulation material is not involved in the fault, this characteristic parameter could not reflect the overall regularity and should not be used^36^.To build a universal model, which must be capable of covering both situations that organic insulating materials are involved or not, the linear shrinking is adopted as the compromise. Meanwhile, the reduced scale is set to 40, which is the result of data fitting. This adjustment can reduce the data scale without affecting the overall regularity. For the characteristic parameter CF_4_/CO_2_, the logarithmic coordinate is also used, while an offset has been added. This is because there are samples with the CF_4_/CO_2_ value much less than 1. Using logarithmic coordinates alone will shrink this parameter value, and adding offsets can avoid it.

5 $${g}_{1}:\mathrm{y}=({SO}_{2}+{SO}_{2}{F}_{2})/40({CF}_{4}+{CO}_{2})$$

6 $${g}_{2}:x={log}_{10}(\frac{{CF}_{4}}{{CO}_{2}}+1)$$

After the pretreatment, six machine learning methods were also used in the sample training of this model. Two of them were chosen to build highly recognizable models. Considering the increase of parameters and classified objects, contour lines were used to present the model results. Finally, Gaussian Distribution was selected to describe the type of insulation defect of the discharge fault and tested by the testing dataset prepared before.

## Results and discussion

**Figure 3:**
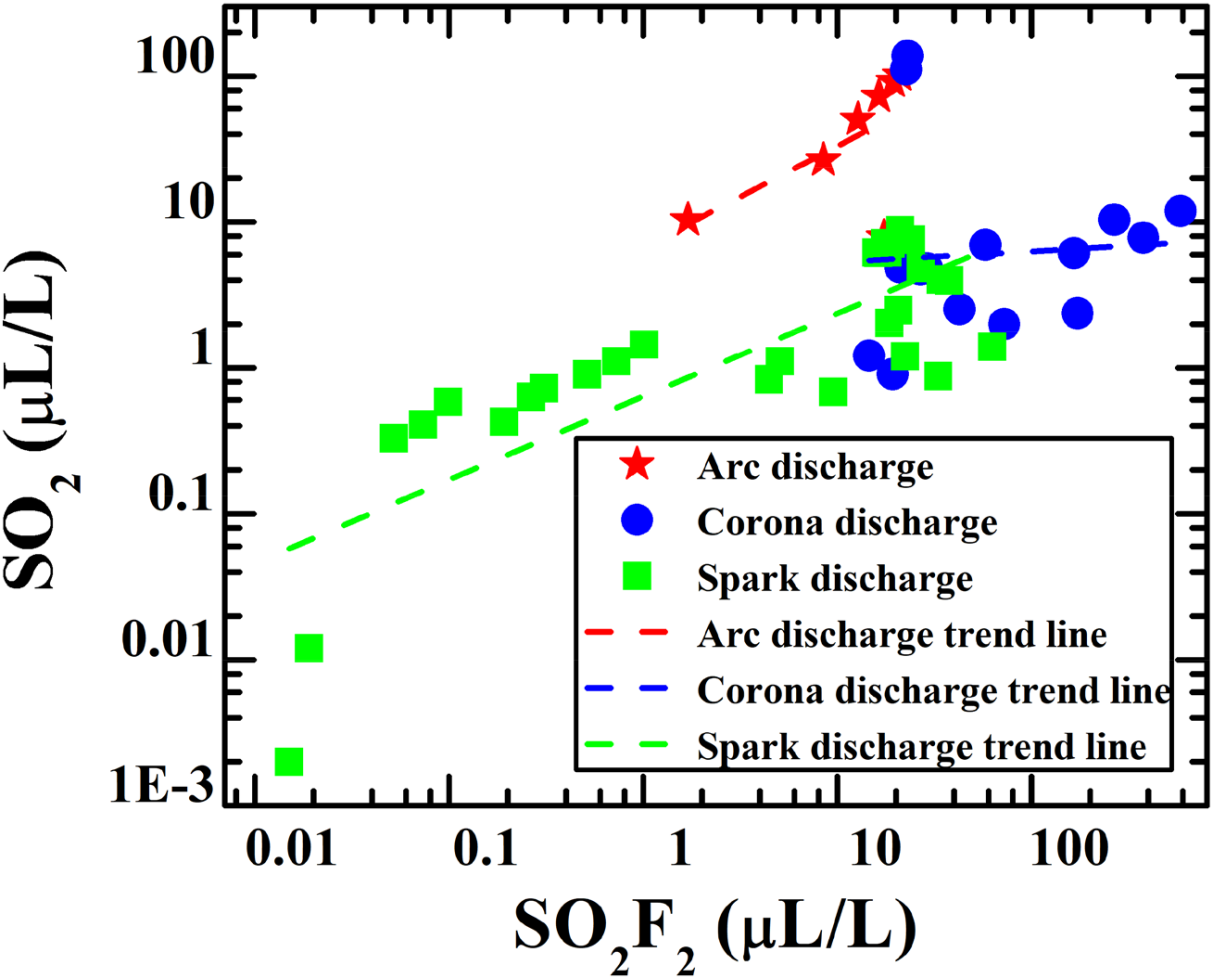
Relative distribution relationship between the SO_2_, SO_2_F_2_ concentration, and discharge fault types (arc, corona, and spark discharge). The linear fitting results are used to guide the visualizations.

The SF_6_ decomposition component data as mapped in Fig. 3 are the sample points for training the fault discharge severity model. The sample point set of each discharge fault is distributed in a certain area. From the linear fitting results, the corresponding slope values of different areas are also different. Arc discharge has the biggest slope and the slope of spark discharge is smaller, while the slope of corona discharge is the smallest. For different types of discharge, SO_2_F_2_/SO_2_ exhibits an aggregation distribution at different centers. This indicates that there is a strong correlation between the concentration ratio of decomposition product SO_2_F_2_/SO_2_ and the discharge fault types. Furthermore, this means that the concentration ratio of SO_2_F_2_/SO_2_ can be used to distinguish the discharge fault type with a different energy.

**Figure 4:**
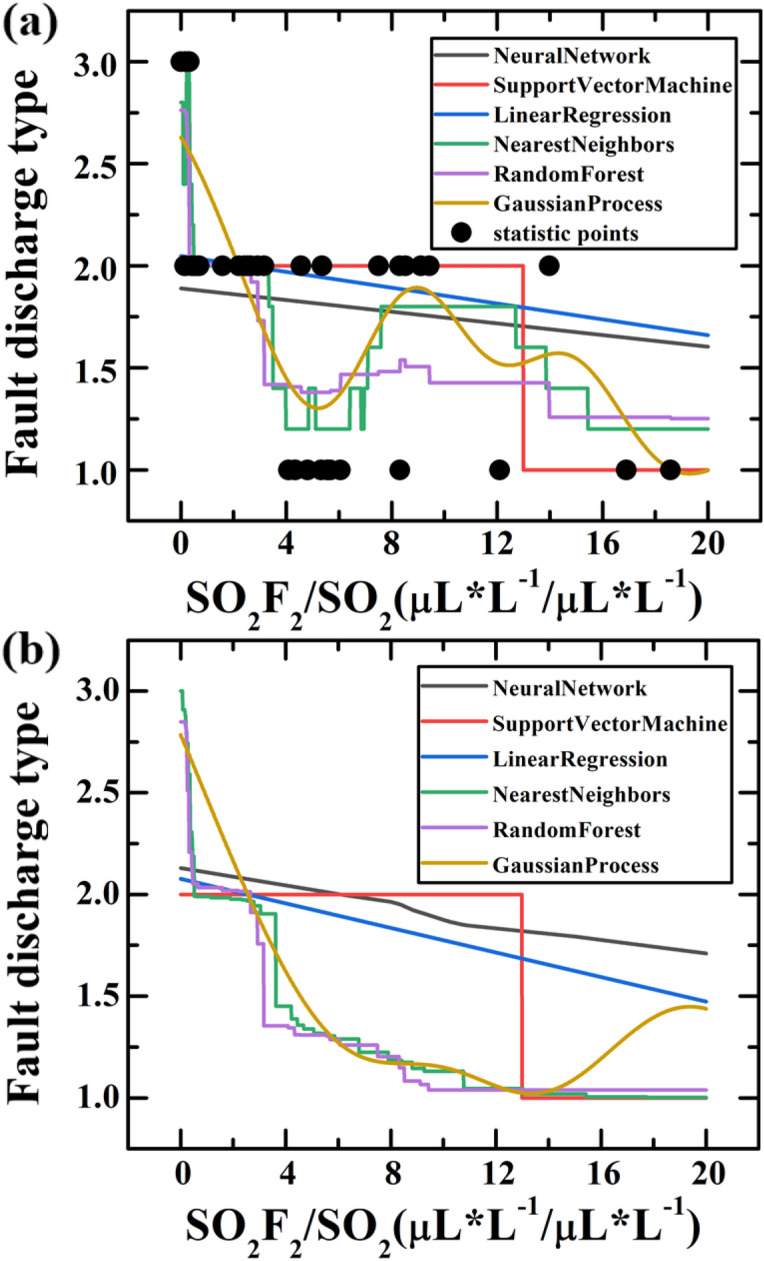
(**a**) The relationship between fault discharge type and the concentration ratio of SO_2_F_2_/SO_2_ calculated by direct machine learning. (**b**) The relationship between fault discharge type and the concentration ratio of SO_2_F_2_/SO_2_ calculated by the machine learning with adjusted data, which can well describe the energy of the discharge fault.

Figure 4a shows the relationship between discharge fault type and the concentration ratio of SO_2_F_2_/SO_2_ calculated by direct machine learning based on six different algorithms. It cannot give a good model to describe the discharge fault type. The reason is that there is a clear boundary between the two discharge fault states, especially the sudden change of the SVM model between 12 and 14. Moreover, the coexistence of two discharge fault types in GIS equipment was ignored and the data has not been preprocessed into a probabilistic form. With the discharge energy increasing, the discharge fault type will experience five states: corona discharge, corona-spark discharge coexistence, spark discharge, spark-arc discharge coexistence, and arc discharge. This process is continuous and progressive, while the discharge fault types corresponding to the component data are discrete. The non-correspondence between the two led to poor machine learning results.

Therefore, the original data need to be preprocessed with different weights. This weight adjustment is aimed to reduce the probability of sample points appearing in the edge area, so that the samples which being unstable (both two discharge state exist in this area) in the overlapping area of the two discharge types in Fig. 4a would tend to be unified. It will facilitate machine learning to fit a suitable curve model to match the distribution of data and the true chemical process. Therefore, it is necessary to establish a continuous function correspondence between the concentration ratio of SO_2_F_2_/SO_2_ and the discharge fault energy: in general, the three empirical probability functions (2)–(4) are mainly based on data fitting and chemical kinetics analysis, as a process of extracting new features through statistics, transformation and operation in machine learning. The empirical probability of arc discharge can be described by a function that drops sharply in the range of 0–0.5. Since the decline is very steep, the form of the function may not be unique. The SO_2_F_2_/SO_2_ ratio of corona discharge is high in general, and the probability has a significant increase at the ratio of about 4. Given that the chemical reaction rate usually satisfies the Arrhenius model, it was originally expressed by:

7 $$\partial M/\partial t={A}_{0}{e}^{-\frac{\Delta E}{{k}_{B}T}}$$

where $M$ is the amount of the reactant; $\partial M/\partial t$ represents the reaction rate of the temperature at T (thermodynamic temperature); ${k}_{B}$ is the Boltzmann constant; $T$ is the absolute temperature; ${A}_{0}$ is a constant; $t$ is the reaction time; $\Delta E$ (eV) is the activation energy of the reaction, which is constant for the same reactant of the same chemical reaction. If the amount of the initial state reactant is ${M}_{1}$, the corresponding reaction time is ${t}_{1}$; the other state is ${M}_{2}$, and the corresponding time is ${t}_{2}$. In the case of temperature $T$ being constant, the cumulative digestion amount from ${t}_{1}$ to ${t}_{2}$ can be given by

8 $${M}_{2}-{M}_{1}={\int }_{{t}_{1}}^{{t}_{2}}{e}^{-\frac{\Delta E}{{k}_{B}T}}dt={e}^{-\frac{\Delta E}{{k}_{B}T}}\Delta t$$

Function (8) indicates that the consumption of reactants or the accumulation of products has an exponential relationship with respect to the activation energy $\Delta E$ of the reaction. Also, the data characteristics are illustrated in Fig. 3 that the slope value $\mathit{log}({n}_{{so}_{2}})/log({n}_{{so}_{2}{F}_{2}})$ of the linear cluster of fault discharges is constant. As referred to the Arrhenius model and the data characteristics, the s-shaped ${f}_{2}(x)$ that rises sharply at the ratio interval of 4–5 was chosen as the empirical probability function for corona discharge. For spark discharge, the empirical probability function can be derived from the normalization principle.

Figure 4b shows the results of re-machine learning obtained by adjustment of the empirical probability functions. It can be found that KNN and the Random Forest algorithm were more easy and suitable, both of them presented an obvious and gentle gradient step shape, which is more realistic. For the KNN-based machine learning algorithm suitable for the modeling in this situation, the explanation can be that: (1) the KNN algorithm mainly relies on the surrounding limited samples, rather than discriminating the sample's overall class domain to determine the category. Therefore, for the sample set divided with more overlapping domains, the KNN method not only is suitable for classification, but it also has a strong transition compared with other algorithms, which is in good agreement with our empirical probability model. (2) The KNN algorithm has the characteristics of high precision and insensitivity to outliers with the weighted average of different distances. It is suitable for classifying rare events, which is consistent with the GIS equipment discharge faults in our analysis object.

**Figure 5:**
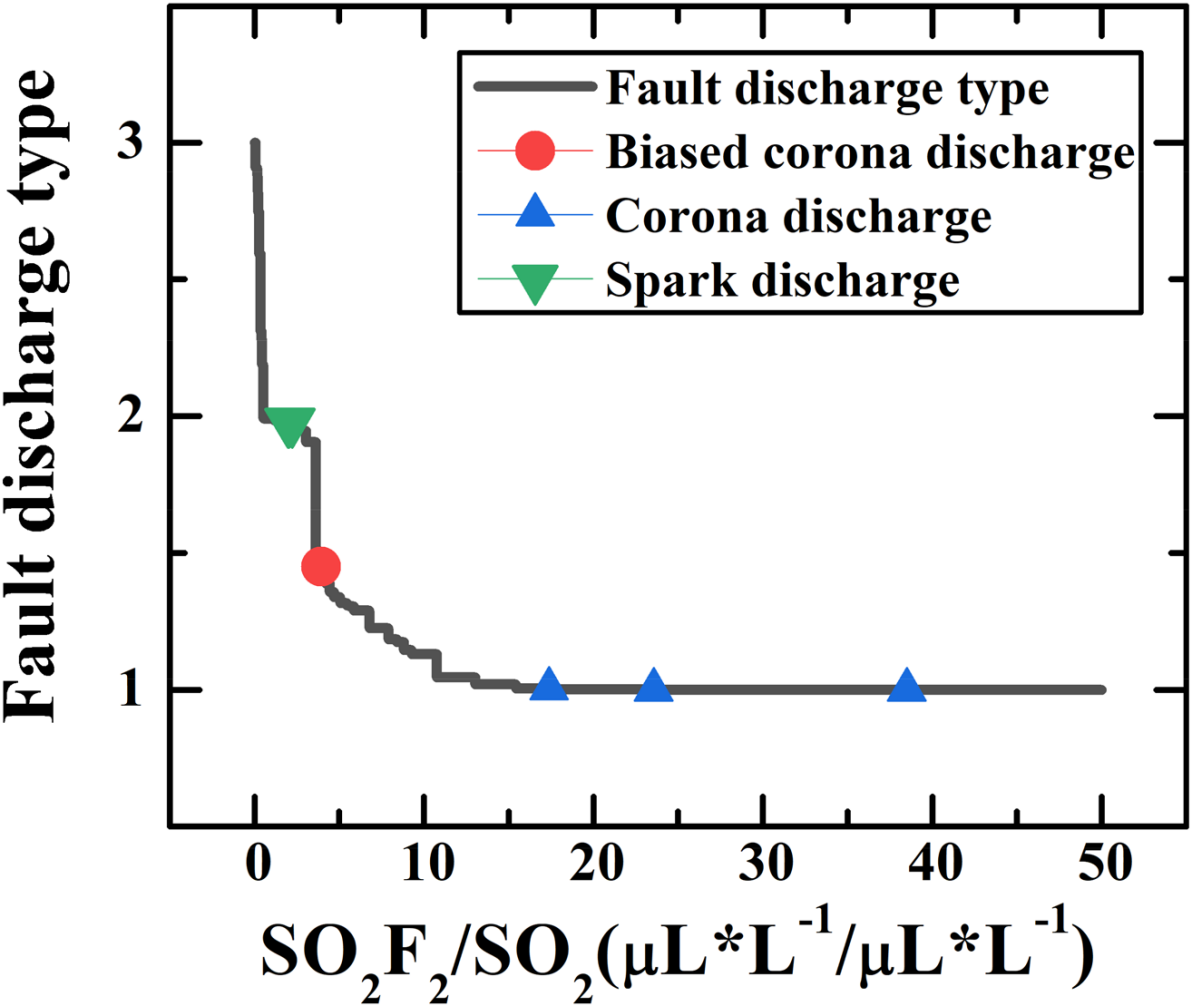
The identification model for severity of discharge fault trained by KNN method under characteristic parameter SO_2_F_2_/SO_2_ and the result of the test points.

Figure 5 shows the test results for the discharge fault severity model trained by KNN. The three types of discharge fault follow a step-shaped distribution of energy levels. In arc discharge area, the value of SO_2_F_2_/SO_2_ ranges from 0 to 0.4. In the spark discharge area, it ranges from 0.4 to 4.2 and the value of SO_2_F_2_/SO_2_ is bigger than 10 in the corona discharge area. It's worth noting that the dot between spark discharge and corona discharge gives a predicted value below 1.5, which means that it is dominated by corona discharge. Based on this, the gradual change of SO_2_F_2_/SO_2_ from 4.2 to 10 illustrates the biased corona discharge state, a transition state from corona discharge to spark discharge.

Furthermore, the decomposition process of SF_6_ under different discharge types can be predicted in this model. In the initial corona discharge, a small amount of S–F bond is broken and react with trace oxygen in the reaction zone to produce low fluoride (SO_2_F_2_ mainly). In a biased corona discharge state, the S–F bond breaks further but very slowly. When the concentration ratio of SO_2_F_2_/SO_2_ is greater than the critical point at 4.2, the discharge fault enters a high energy state with a large number of S–F bond breaking. Because of the existence of oxygen in the reaction zone, the concentration of SO_2_ increases rapidly, and the concentration ratio of SO_2_F_2_/SO_2_ decreases with the increase of energy. It can be inferred that SF_6_ has a very large fracture degree of S-F bond under high-energy discharge, and the fracture speed becomes much faster after the biased corona discharge state^21^. Based on this, the point at 4.2 can be used as a critical point between high and low energy discharge states, which is of great significance for high energy discharge fault warning. In addition, it was also found that the point SO_2_F_2_/SO_2_ = 1 falls in the span of spark discharge. This characteristic can be used to explain the inability to compare the content relationship between SO_2_ and SO_2_F_2_ in spark discharge^26,37^.

**Figure 6:**
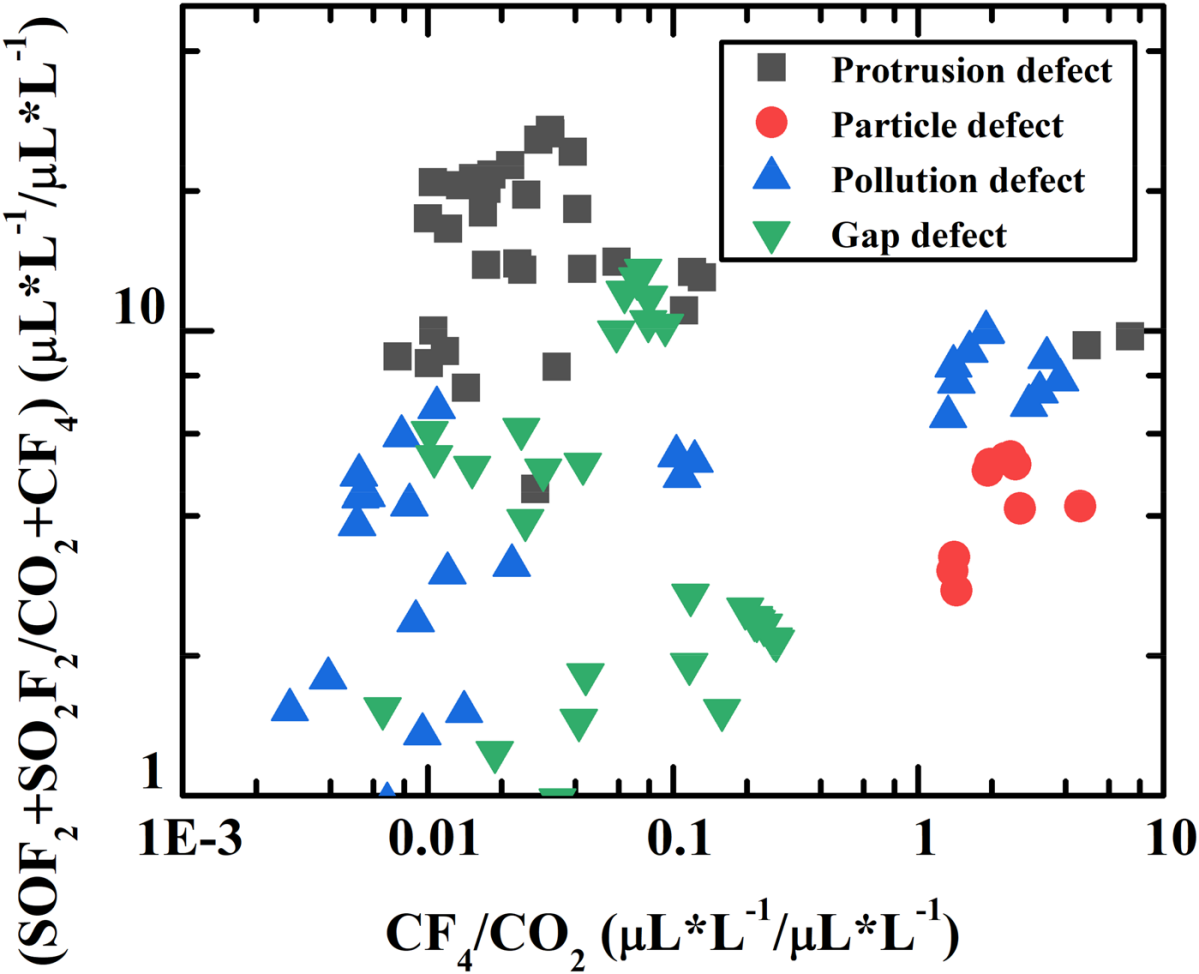
The combination of (SO_2_ + SO_2_F_2_)/(CF_4_ + CO_2_) and CF_4_/CO_2_ performs well because of the discrete characteristic except for the conflict between the pollution defect and gap defect.

**Figure 7:**
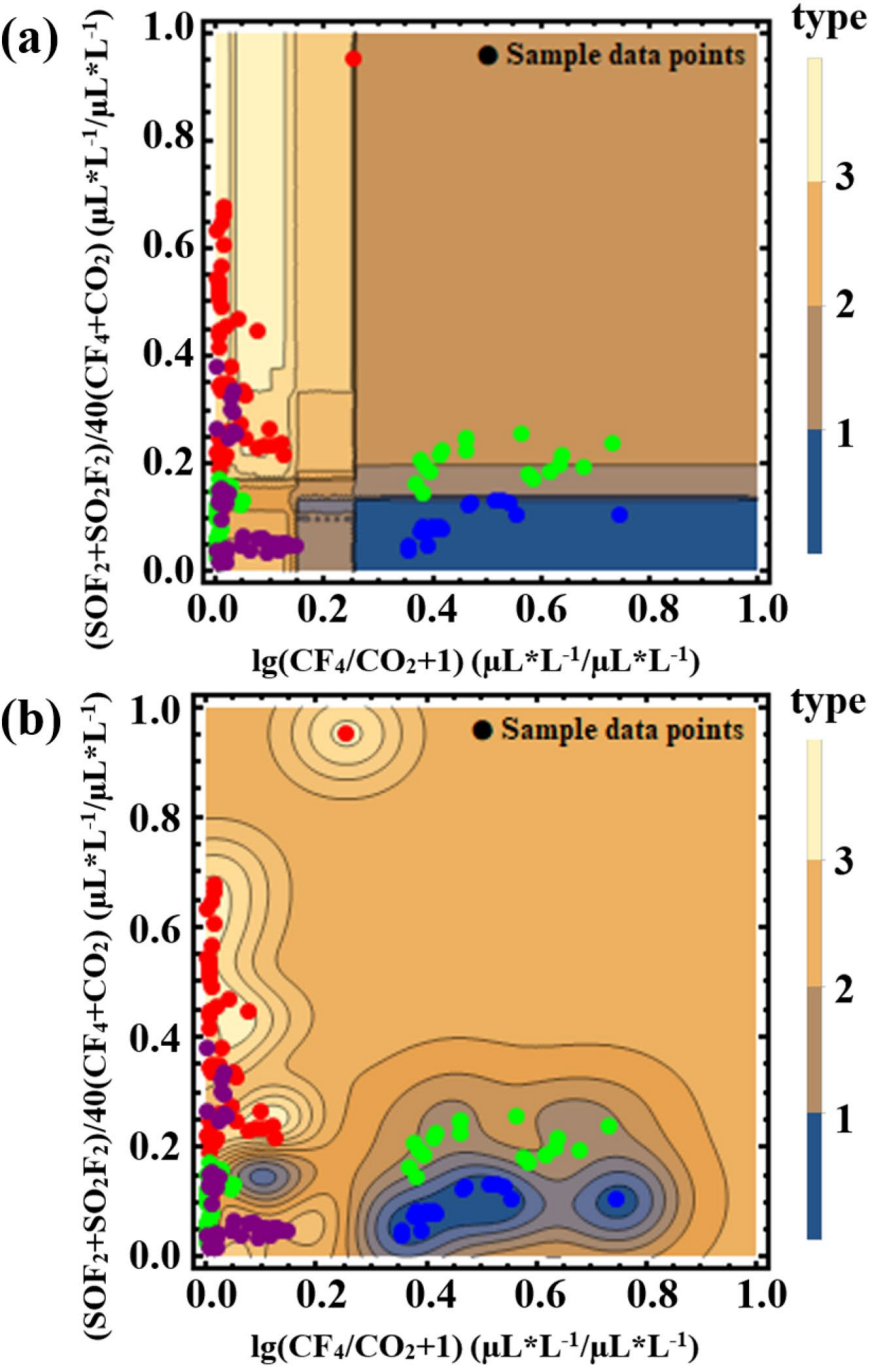
(**a**) The relationship between insulation defects of fault discharge and the characteristic parameters (SO_2_ + SO_2_F_2_)/(CF_4_ + CO_2_) and CF_4_/CO_2_ calculated by the Random Forest method with the contour model. (**b**) The relationship between insulation defects of fault discharge and the characteristic parameters (SO_2_ + SO_2_F_2_)/(CF_4_ + CO_2_) and CF_4_/CO_2_ calculated by the Gaussian Distribution method with the contour model.

Figure 6 shows the sample data organized with the parameters CF_4_/CO_2_ and (SOF_2_ + SO_2_F_2_)/(CF_4_ + CO_2_). A certain aggregation and continuity of the distribution of insulation defect types for each discharge fault can be discovered. Figure 7 gives the two better models from the six: random forest distribution and Gaussian Distribution. The parallel line boundary of the Random Forest model is simple and effective in the fault judgment, while the Gaussian Distribution model has a good smooth slope and fits well with the actual data distribution. For the high conformity of Gaussian Distribution in this application scenario, the explanation can be considered: when the data presents a nonlinear trend, the Gaussian process regression can be combined with the Bayesian probability algorithm to give the probability of the predicted value and the confidence interval in the form of multidimensional Gaussian Distribution. This non-linear classification property and the idea of ​​considering the sample probability distribution are in good agreement with the probability model.

**Figure 8:**
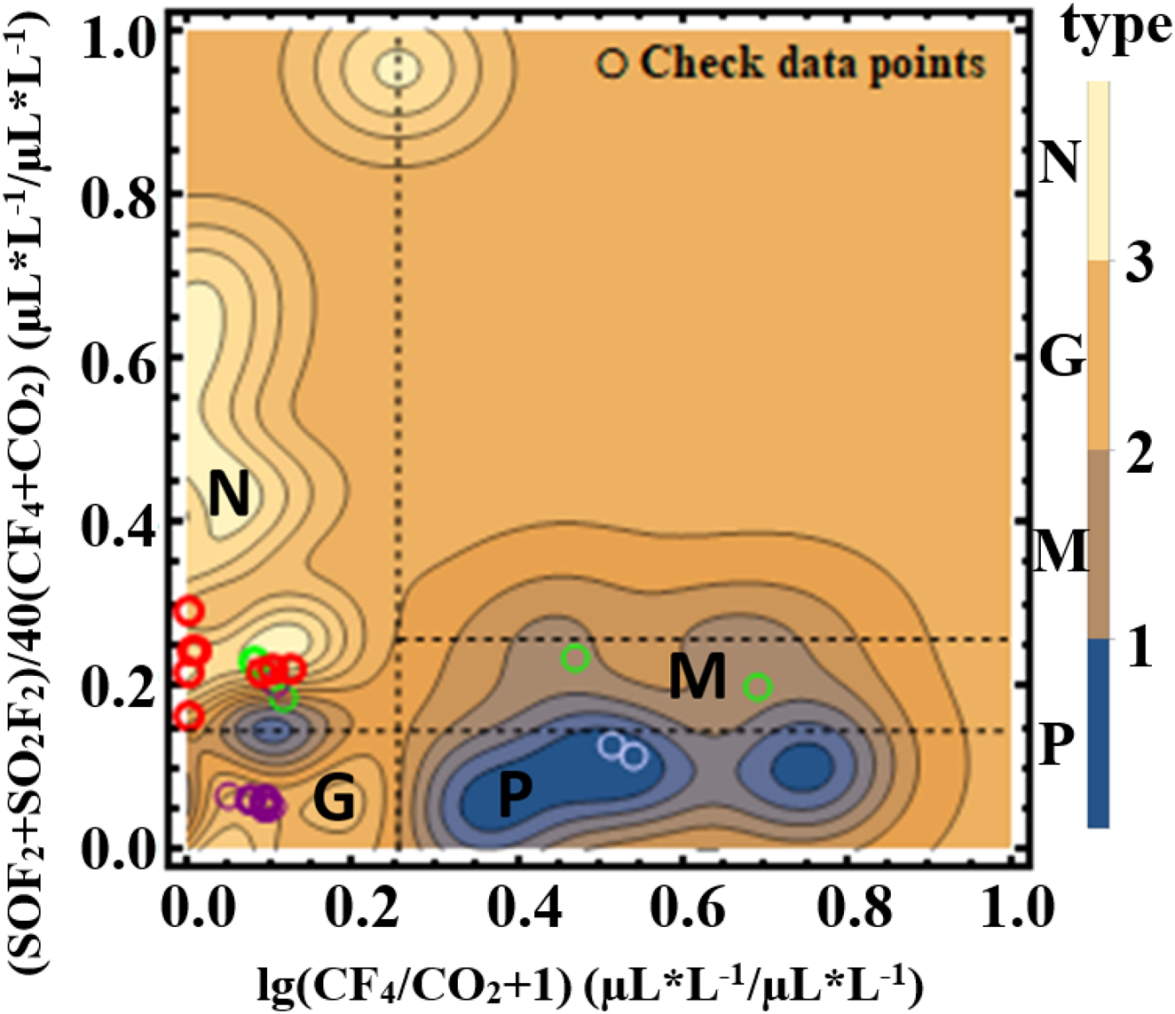
The identification model for insulation defects of fault discharge trained by the Gaussian Distribution method under characteristic parameters (SO_2_ + SO_2_F_2_)/(CF_4_ + CO_2_) and CF_4_/CO_2_ with the result of the test points.

Figure 8 shows the test results for the insulation defect model obtained by Gaussian Distribution. It can be roughly seen that four kinds of insulation defects have their clustering areas, which are labeled as N (protrusion defect), G (gap defect), M (pollution defect), and P (particle defect) respectively. Compared with the results (the hidden lines showed between different clustering) of the code tree method, it can be seen that the classification of insulation defects is consistent, except for some pollution defects in region N and G^38^. The pollution defects near the boundary between region N and region G, where (SOF_2_ + SO_2_F_2_)/40(CF_4_ + CO_2_) ≈ 0.15, are interpreted as insulator surface pollution defects which are caused by secondary effects of other defects^21^. In this area, the low value of CF_4_/CO_2_ shows that F atoms produced by the fracture of the S–F bond react more with the metal, which reduces the amount of CF_4_ and leads to the severe deterioration in metal. While the value of (SOF_2_ + SO_2_F_2_)/40(CF_4_ + CO_2_) changes a lot from gap defect to protrusion defect. Considering the same decomposition degree of SF_6_, the value of (SOF_2_ + SO_2_F_2_)/40(CF_4_ + CO_2_) mainly depends on the number of carbon atoms from the degraded organic insulation. As a result, the insulator surface pollution defects are caused by secondary effects of gap defect with less degraded organic insulation or protrusion defect with more degraded organic insulation. Such an effect means that the coexistence of multiple insulations, which is worth noting during the GIS maintenance. By revealing the surface insulator pollution defects which code tree method does not have, this model explains why some identification models misjudge at the boundary^39^. In addition, the pollution defects in the M region represents internal insulator pollution defects. The model was also compared with models with more characteristic parameters^34^. It can be concluded that, even with the addition of the SOF_2_/SO_2_F_2_ characteristic parameter, the sample points of the pollution defect of insulation still overlap with other defects. This comparison proves the existence of multiple discharges and supports the feasibility of using only two parameters CF_4_/CO_2_ and (SOF_2_ + SO_2_F_2_)/(CF_4_ + CO_2_).

## Conclusions

In general, a well-trained machine learning model obtained by optimized data from the different environments can describe the severity of fault discharge and the type of insulation defect after pre-processing. In the severity model obtained by KNN, a biased corona discharge state is revealed as a transition state from corona discharge to spark discharge, where SO_2_F_2_/SO_2_ ranges from 4.2 to 10. Meanwhile, the critical point at 4.2 is of great significance for high energy discharge fault warning, because of the rapid fracture of S–F bond and energy increase after this point. In the insulation defect model obtained by Gaussian Distribution, the region near the boundary, where (SOF_2_ + SO_2_F_2_)/40(CF_4_ + CO_2_) ≈ 0.15, is considered as a multiple discharge coexistence area. In this area, surface insulator pollution defects will be caused by secondary effects of gap defect with less degraded organic insulation or protrusion defect with more degraded organic insulation. Such an effect is worth noting during GIS maintenance. Considering the data from a different environment and preprocessing the data uniformly, this model reveals some states not mentioned before. In addition, some improvement measures can help our model performs better in the application, such as more sample data of real added, more possible insulation defects considered, and clearer decomposition mechanism of SF_6_ discussed. In this case, the probability model would be more reliable on the evaluation of the fault of the GIS system.
